# Enhancer-promoter communication: unraveling enhancer strength and positioning within a given topologically associating domain (TAD)

**DOI:** 10.1038/s41392-022-01114-8

**Published:** 2022-08-12

**Authors:** Benedetto Daniele Giaimo, Tilman Borggrefe

**Affiliations:** Institute of Biochemistry, Justus-Liebig-University Giessen, Friedrichstrasse 24, 35392 Giessen, Germany

**Keywords:** Epigenetics, Biochemistry

In a recent study published in *Nature*, Zuin et al. elucidate the molecular mechanisms of how enhancer positioning and strength affect gene expression levels.^1^

Cell-type-specific transcription factors (TFs) and ubiquitously expressed cofactors regulate the complex network of eukaryotic gene transcription in an exquisitely specific and sensitive manner. The DNA elements bound by TFs are called enhancers and can be located close to the transcription start site (TSS) or far away, even hundreds of kilobases (kb) away. Historically, enhancers were identified in the early 1980s by injecting DNA into nuclei or cells.^2^ Subsequently, several assays such as EMSAs (electrophoretic mobility shift assays) and DNAse footprinting analyses were used to identify enhancers which were further functionally characterized by luciferase assays. These techniques have been key in discovering and characterizing many different enhancers and their cognate TFs. In the last two decades genome-wide techniques like ATAC-Seq (assay for transposase-accessible chromatin using sequencing), ChIP-Seq (chromatin immunoprecipitation followed by sequencing) combined with RNA-Seq widened our understanding of enhancer biology. Still the long-standing questions remained: What are the principles governing the enhancer-promoter communication and what are the key regulatory steps resulting in adequate transcriptional output? Does genomic context and possible insulation matter? In this regard, Chromatin Conformation Capture (3C) and related techniques^3^ significantly enlarged our understanding of enhancer biology. Topological associating domains (TADs) were defined as the building blocks of genome organization, in which enhancers operate.^4^ The relationship between enhancers and TADs was and is still intensely studied and whole novel toolbox of techniques is available to study enhancer functions.^5^

In this context, Zuin et al. developed an unbiased experimental strategy in mouse embryonic stem cells (ESCs) to investigate how genomic distance between a given enhancer element and its cognate TSS affects transcriptional output within a specific TAD.^1^ For this purpose, a transgene was inserted on chromosome 15 within a TAD that does not contain any gene or active enhancer. In particular, this region of ~500 kb in size was chosen because it is “neutral” and its structural complexity is minimal. The transgene carries the mouse *Sry* (*sex determining region Y*)-*box 2* (*Sox2*) promoter that drives the expression of the enhanced green fluorescent protein (eGFP). The eGFP is divided into two parts by a *piggyBac* transposon that contains the cognate enhancer of the *Sox2* promoter known as *Sox2* control region (SCR). Enhancer hopping only of the SCR is mediated via expression of the *piggyBac* transposase (PBase), that leads to excision and reintegration of the enhancer randomly in *cis* in the vicinity of the original site This system allowed Zuin et al. to generate hundreds of individual clones or cell lines, each having the very same enhancer element positioned in different locations within a given TAD.

By using this system, Zuin et al. could observe that gene expression levels rapidly decrease with increasing distance between the enhancer and the promoter, as measured on protein level by eGFP intensity or mRNA number per cell using RNA-FISH (RNA-fluorescence in situ hybridization) (Fig. 1a). In addition, there is a nonlinear correlation between gene expression and contact probability; this is also supported by a mathematical two-state model, in which the promoter on rate follows a sigmoidal function of enhancer-promoter contact probability (Fig. 1b). Importantly, there is an interplay between enhancer and insulators: Strong enhancers are less susceptible to insulation by CTCF compared to weak enhancers (Fig. 1c). The chosen approach by Zuin et al. is elegant and unbiased and allows the comparison of enhancer positioning in the genomic context with minimal variables.

**Fig. 1:**
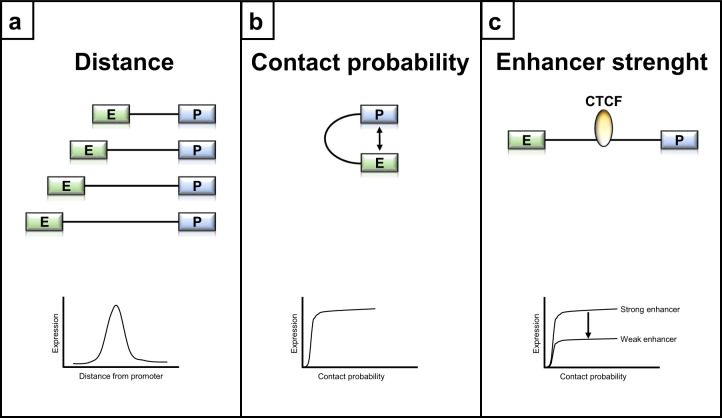
Schematic summary of the main findings of Zuin et al. **a** Gene expression depends on the enhancer-promoter distance. **b** Gene expression depends on contact probability by following a nonlinear relationship. **c** Enhancer strength determines its sensitivity to CTCF-mediated insulation

In future, using such an extremely well-defined system, it will be interesting to investigate how a particular chromatin configuration influences enhancer strength within a given TAD. Would it be possible to predict the outcome of transcriptional output with a mathematical model? And again, what are the critical variables in such an equation? Would it be also possible to define the differences between a locus with multiple enhancer elements, such as super-enhancers? In this case, are the rules different?

Taken together, the study from Zuin et al. reveals that changes in frequency of promoter bursting dynamics is nonlinear and depends on contact probability, enhancer strength, and positioning in relation to boundary elements. These findings broaden our fundamental understanding of enhancers in a genome-wide context.

## Supplementary information

Checklist

## References

[1] Zuin J (2022). Nonlinear control of transcription through enhancer-promoter interactions. Nature.

[2] Grosveld F, van Staalduinen J, Stadhouders R (2021). Transcriptional regulation by (super)enhancers: from discovery to mechanisms. Annu. Rev. Genomics Hum. Genet..

[3] Kempfer R, Pombo A (2020). Methods for mapping 3D chromosome architecture. Nat. Rev. Genet..

[4] Ciabrelli F, Cavalli G (2015). Chromatin-driven behavior of topologically associating domains. J. Mol. Biol..

[5] Giaimo BD, Friedrich T, Borggrefe T (2021). A comprehensive toolbox to analyze enhancer-promoter functions. Methods Mol. Biol..

