# Supplementary material for: Enhancer-promoter communication: unraveling enhancer strength and positioning within a given topologically associating domain (TAD)
Source: Signal Transduct Target Ther. 2022 Aug 12;7:281. doi: 10.1038/s41392-022-01114-8 (PMC9374741; doi:10.1038/s41392-022-01114-8)
Supplement: Supplementary file 1 — Checklist [file 41392_2022_1114_MOESM1_ESM.pdf]

Author Checklist before final submission for Research Highlight (STTT)

☑ **Words** (1,000 words max excluding references and figure legend).

☑ **Authorship** (no more than 3 authors).

☑ **Author institutions or affiliations** (as concise as possible).

☑ **DO NOT** put the first one or two sentences (the brief introduction) into bold font.

☑ **Figure:** Max of 1; should be uploaded as a single file.

☑ **Image** resolution of **at least 300 dpi** at publication size.

☑ **Figures** divided into parts should be labelled with a **lower-case**, **boldface** 'a', 'b', etc.in the **top left-hand** corner.

☑ **Figures** use the same typeface (Arial or Helvetica) for all figures. Use symbol font for Greek letters.

☑ **Figures** are best prepared at the size you would expect them to appear in print. At this size, the optimum font size is 8pt and no lines should be thinner than 0.25pt (0.09 mm).

☑ **Figures** (or parts of figures) generated from online tools (e.g., **BioRender, etc**) or including online picture resources (e.g. from **SMART - Servier Medical ART, etc**) should be clearly indicated by **citing the original websites in the figure legend** or following the requirements mentioned in the original websites.

☑ **Reference style** (All authors should be listed for papers with up to 5 authors; for papers with more than 5 authors, the first only should be listed, followed by et al. Abbreviations for titles of medical periodicals should conform to those used in the latest edition of Index Medicus. The first and last page numbers for each reference should be provided. References to the literature in the text should be typed as **superscripts after any punctuation**.)

☑ **Reference number:** Max of 5.

☑ When describing COVID-19-associated background and results, please **avoid using** the words such as "Wuhan virus/strain", "Hubei", "spread from China", etc.

☑ **Figure style** (Please refer to Example 1 and 2)
